# A delicate balance between rejection and BK polyomavirus associated nephropathy; A retrospective cohort study in renal transplant recipients

**DOI:** 10.1371/journal.pone.0178801

**Published:** 2017-06-13

**Authors:** Lilli Gard, Willem van Doesum, Hubert G. M. Niesters, Willem J. van Son, Arjan Diepstra, Coen A. Stegeman, Henk Groen, Annelies Riezebos-Brilman, Jan Stephan Sanders

**Affiliations:** 1 Department of Medical Microbiology, University of Groningen, University Medical Centre Groningen, Groningen, The Netherlands; 2 Department of Internal Medicine, Division of Nephrology, University of Groningen, University Medical Centre Groningen, Groningen, The Netherlands; 3 Department of Pathology, University of Groningen, University Medical Centre Groningen, Groningen, The Netherlands; 4 Department of Epidemiology, University of Groningen, University Medical Centre Groningen, Groningen, The Netherlands; 5 Department of Medical Microbiology, University Medical Centre Utrecht, Utrecht, The Netherlands; Ohio State University Wexner Medical Center, UNITED STATES

## Abstract

**Background:**

The immunosuppressive agents mycophenolate acid (MPA) and tacrolimus (Tac) are associated with a higher incidence of BK polyomavirus nephropathy (BKPyVAN).

In this observational retrospective cohort study, the frequency of BK polyomavirus (BKPyV) complications over a 24-month period was studied.

**Methods:**

358 renal transplant recipients (RTR) treated with MPA, with either cyclosporine A (CsA) (CsAM group) or Tac (TacM group) and mostly prednisolone, were included.

**Results:**

Incidence of BKPyV-viremia was not significantly different between the CsAM (n = 42/191) (22.0%) and the TacM (n = 36/167) (21.6%) group. Biopsy proven BKPyVAN occurred more often in the TacM group (6.6%) versus the CsAM group (2.1%) (p = 0.03). Longitudinal data analysis showed a significant earlier decline of viral load in plasma in the CsAM group compared to the TacM group (p = 0.005).

The incidence of biopsy proven acute rejection (BPAR) was significantly higher in the CsAM (19.9%) compared to the TacM (10.8%) (p = 0.02) group. Graft loss, estimated glomerular filtration rate and mortality rate did not differ in both treatment groups.

**Conclusion:**

In conclusion, this study shows that immunosuppressive treatment with Tac and MPA compared to CsA and MPA is associated with a lower incidence of BPAR, but at the cost of an increased risk of developing BKPyVAN in the first two years post-transplant.

## Introduction

Increasing rates of BK polyomavirus nephropathy (BKPyVAN) have been observed in renal transplant recipients (RTR) over the last decades. Recent data suggest that the risk of BKPyV related pathology results from both pre- and post-transplant factors [1–5]. Multiple studies identified tacrolimus (Tac) as a risk factor for developing BKPyV viremia or BKPyVAN [1,2,6–8]. In contrast, cyclosporine A (CsA) seems to suppress BKPyV replication *in-vitro* [9], but it is unclear whether this antiviral effect is present in RTR. This suggests that the choice of immunosuppression plays a role in the increasing cases of BKPyVAN in the last decades, since most RTR receive immune suppressive therapy with Tac after renal transplantation [10,11].

Data from a study of 682 RTR treated with either Tac or CsA with mycophenolate sodium (MPS) showed that there was a significant difference between the two groups at 6 and 12 months for the rate of BKPyV-viremia [1]. No other large studies have investigated the occurrence of BKPyV related complications in RTR, comparing different treatment regimens.

We aim to further clarify the clinical impact of different immunosuppressive regimens on the occurrence of BKPyVAN after renal transplantation.

In this observational retrospective cohort study, in 358 consecutive RTR treated with mycophenolic acid (MPA) with either CsA or Tac, the risk of BKPyV-viremia and BKPyVAN over a 24 months’ period was studied.

## Material and methods

### Patients

**Fig 1:**
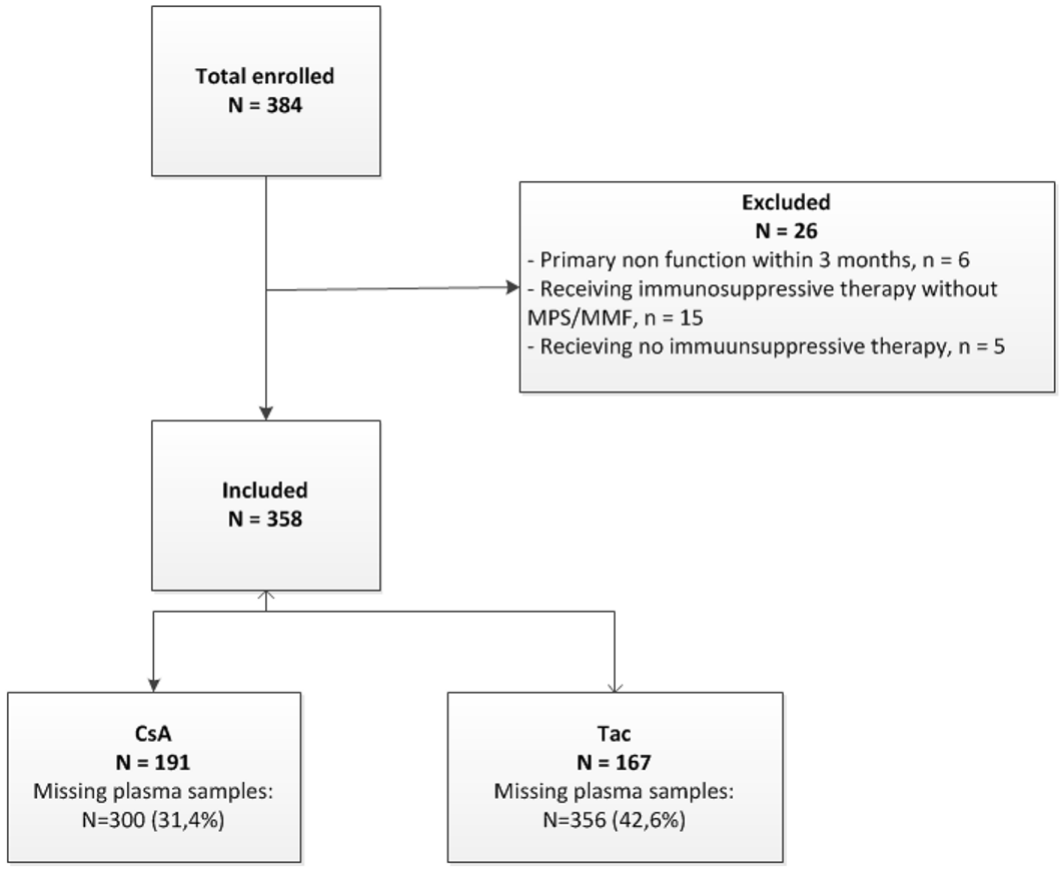
Flowchart study population. Overview of included and excluded recipients. CsAM: cyclosporine A with or without prednisone and MPS or MMF, TacM: tacrolimus with or without prednisone and MPS or MMF.

Patients that underwent a first, second or third renal transplant between January 2010 and December 2012 at the University Medical Center Groningen (UMCG) were included. Exclusion criteria were primary non-function of the allograft within 3 months, patients receiving no immunosuppressive therapy or receiving immunosuppressive therapy without MPA (Fig 1).

The immunosuppressive regimens consisted of quadruple immunosuppression with basiliximab induction, a calcineurin-inhibitor, i.e. Tac or CsA, MPA, i.e. mycophenolate mofetil (MMF) or MPS and prednisolone. By protocol, RTR received CsA before January 2012 and Tac from January 2012 onwards. Based on clinical indication some recipients received also Tac before January 2012 or steroid-free immunosuppression. MPA was started at 2000 mg/day and adapted to1500 mg/day when combined with tacrolimus, and subsequently 1000 mg/day or 500 mg/day based on clinical indications, especially if side-effects to MPA existed. Immunosuppression was adapted to BKPyV-viremia by first reducing MPA by half and if BKPyV viremia did not decrease within 1 month MPA was stopped and subsequently Tac or CsA was reduced. Anti-rejection therapy consisted of first methylprednisolone intravenously on three consecutive days, if a vascular rejection occurred or in case of steroid resistance rescue therapy with Anti-Thymocyte Globulin (ATG) was started.

The Ethics Committee of the University Medical Center Groningen decided that the study did not fall under the scope of the Medical Research Involving Human Subjects Act ((METc 2015/448)). Therefore, approval by an ethics committee was not indicated for this study, because of the absence of any risk for the participants. In The Netherlands informed consent of study participants is not required in such retrospective studies. And, in this study no informed consent was given by the study participants. Patient data are stored coded in a study-database and extractions from this database can only be made by members of the study team. Overall, the study fulfilled the criteria of the UMCG research code and Dutch Privacy Act.

### Data collection

Retrospective clinical, virological, and renal biopsy data were collected up to 24 months post transplantation, using electronic patient files.

BKPyV-loads in plasma were measured at time points 6 weeks (±2 weeks) and at month 3 (±3 weeks), 6 (±1,5 months), 12 (±3 months) and 24 (±6 months). Viremia was defined if, at least once, in a plasma sample BKPyV-DNA was measured above 2 log10 cp/ml, at or outside the defined time points. Renal function was measured by using the estimated glomerular filtration rate (eGFR) (MDRD formula) at time points 3 (±3 weeks), 6 (±1,5 months), 12 (±3 months) and 24 (±5 months) months.

Renal biopsies were taken according to protocol one year post transplantation, by indication and by viremia (>4log10) in combination with a decrease of renal function. Renal biopsies were scored by a renal pathologist according to the Banff classification [12]. Biopsy proven acute rejection (BPAR) was defined as Banff IA, IB, IIA, IIB or III and proven BKPyVAN as SV40 positive tubules as reported by the pathologist.

Tac trough drug levels (ug/l) and CsA trough drug levels (ug/l) were collected at time points 2 weeks (±2 days), 6 weeks (±2 weeks), 3 (±3 weeks) and 12 months (±3 months).

### BKPyV real-time PCR and BKPyV genotyping

BKPyV-DNA was measured with an internal controlled quantitative *in-house* Real Time PCR (RT-PCR). Samples collected before January 2012 were extracted using the MagNaPure LC (Roche Diagnostics, Germany) and BKPyV-DNA was measured with RT-PCR(Fwd-gcacttttgggggacctagtt, Rev-ctctacagtagcaagggatgcaatt, Pr-6FAM-cagtgtatctgaggctg-MGB). After January 2012 samples were extracted and amplified as previously described by Gard et al.[13]. Both RT-PCR’s were a multiplex with the internal control, seal herpes virus (PhHV) (Fwd-gggcgaatcacagattgaatc, Rev-gcggttccaaacgtaccaa, Pr-CY5-tttttatgtgtccgccaccatctggatc-BHQ1).

All PCR reactions were performed with 20μl DNA and 30μl PCR mix, containing 2x Universal Mastermix (Thermofisher, USA), 5mg/ml bovine serum albumin (Roche Diagnostics, Germany), 300nM primers and 100nM probe and DNAse/RNAse free water (Sigma, The Netherlands). The ABI PRISM 7500 (Life Technologies, USA) was used for the detection and amplification using the following thermal profile: 50°C for 2min, 95°C for 10min followed by 42 cycles of 95°C for 15sec, 60°C for 1min.

The BKPyV main genotype was determined from the first BKPyV positive sample in a patient using the RT-PCR as described previously [13].

### Serology

The antibody-binding assay as developed by Waterboer et. al. [14] was used for the measurement of antibodies against BKPyV. The protocol was described previously [15]. Cut-off values were calculated for each new set of coupled Bio-Plex polystyrene beads, using 51 children aged from 6 to 24 months to determine the seronegative population. A cut-off value of 744 MFI was used for samples from 2010 and a cut-off value of 602 MFI for samples from 2011 and 2012 [16].

### Area under the curve determination of viral BKPyV-load

The area under the curve (AUC) of viral BKPyV-load was determined using plasma BKPyV-loads until 24 months (±6 months) post-transplantation from transplant recipients that had at least one BKPyV-load above 2log10cp/ml. The calculation was done using the formula as described by Schafer et al.[17]. Time points were defined as t_1_, t_2_ etcetera, depending on the number of measurements and the responding BKPyV-loads were designated as y_1_, y_2_,…, y_n_.

### Statistics

Statistical analyses were performed based on the treatment therapy after transplantation using SPSS IBM Statistics 22 (IBM, USA). Baseline characteristics between the two treatment arms were compared using Chi-square test for categorical variables and the independent sample t-test for continuous variables. Chi-square tests were used for the comparison of BKPyV infection and BKPyVAN between the treatment arms at time points 3 and 12 months. Additionally, occurrence ofBKPyV viremia and BKVPyVAN was compared by Chi-square test at time point 3 months and 12 months between Tac and CsA trough levels above and below the median.

Longitudinal analyses were performed using generalized estimating equations (GEE) with an exchangeable correlation matrix and the resulting estimated marginal means (EMM) with 95% confidence intervals were plotted in graphs. The effect of BKPyVAN on the renal function was analyzed using GEE over a time period from t = 3 to t = 24 months.

Cox regression and Kaplan-Meier analysis were used for the comparison of proven BKPyVAN during 24 months, between the two treatment arms. For this analysis only the viremia group was included. The following variables were used for the multivariate analysis: recipient sex and age, donor sex and age, cold ischemia time, type of donor (living, deceased), BPAR, primary disease, HLA mismatch (AB, DR). Figures were plotted using Graphpad Prism 5.01 (GraphPad Software, Inc, USA). P-values <0.05 were considered significant.

## Results

### Baseline characteristics

**Fig 2:**
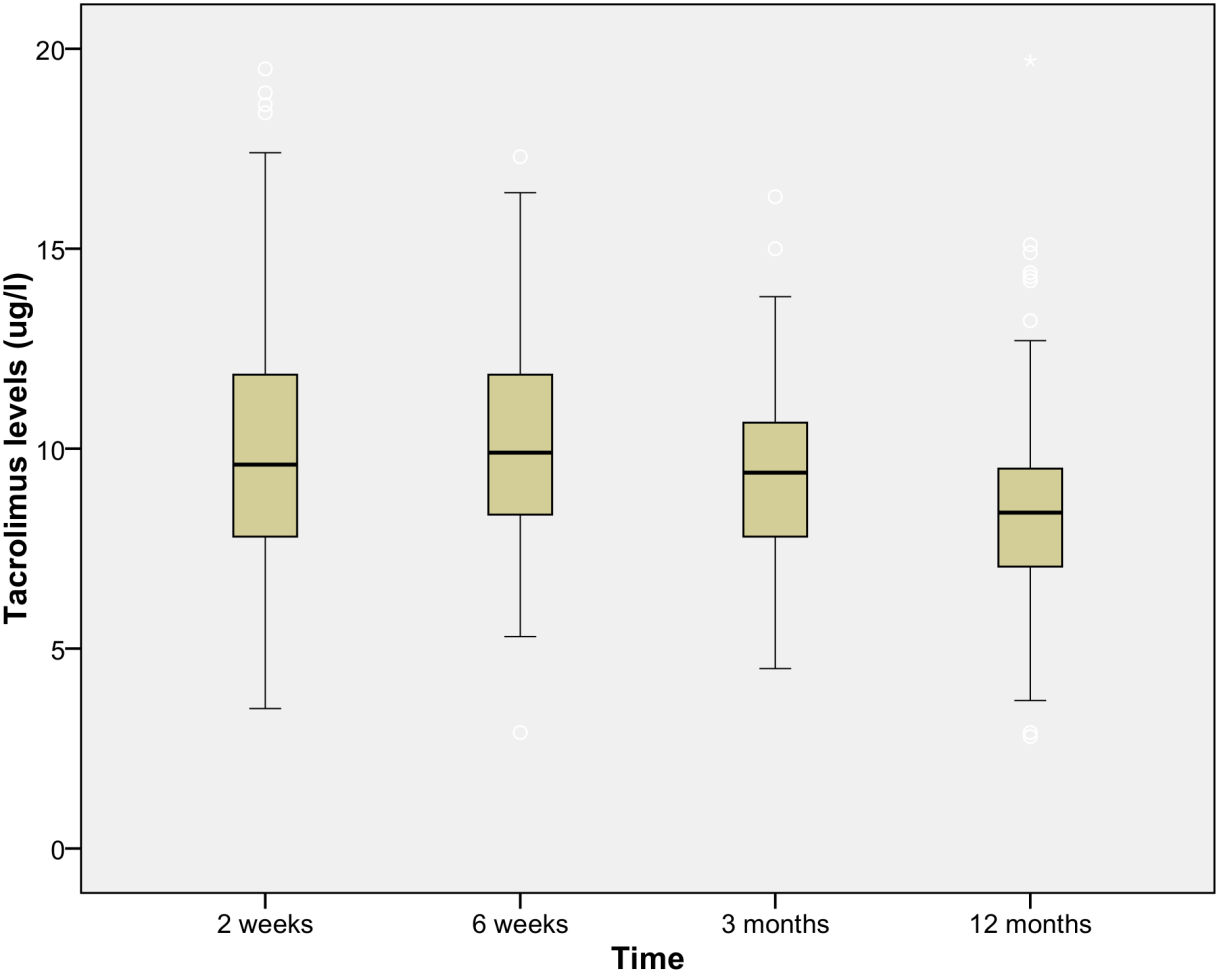
Drug level immunosuppressive Tacrolimus. The tacrolimus levels, mean level at 12 months 8.5ug/l over time in the first year after renal transplantation.

**Fig 3:**
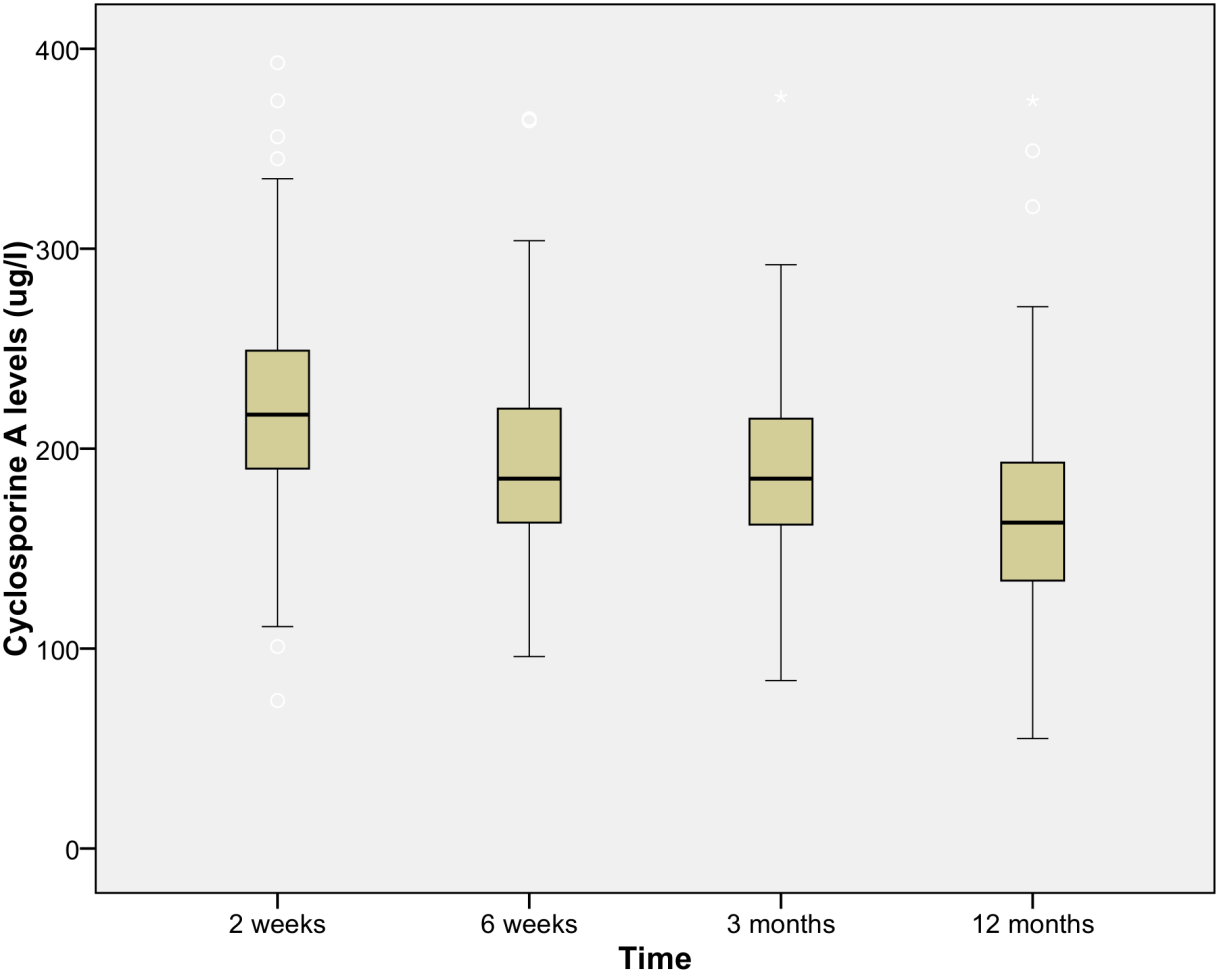
Drug level immunosuppressive Cyclosporine A. Cyclosporine A levels, mean level at 12 months 166.2ug/l over time in the first year after renal transplantation.

From the 384 RTR at the UMCG between 2010 and 2012, 358 were included in the analysis. Based on the treatment directly after transplantation, recipients were divided into two different treatment groups. In the treatment group with CsA and MPA (CsAM group) 190 patients received immunosuppressive therapy with steroids, and one without. The treatment group with Tac and MPA (TacM group) consisted of 161 patients receiving immunosuppressive therapy with steroids, and 6 RTR without. Drug levels for Tac and CsA are depicted in Figs 2 and 3. The median at time point 3 months was respectively 9.55 ug/l and 183.5 ug/l for Tac and CsA and at time point 12 months 8.4 ug/l and 163.5 ug/l. Toxic drug levels for Tac (>20ug/l) and CsA (>400ug/l) were excluded from the analysis.

Baseline characteristics did not differ significantly between the two groups (Table 1), with an exception for distribution of primary disease of the recipient and cold ischemia time of the deceased donor.

**Table 1:** Baseline characteristics by treatment group.

	CsAM (N = 191)	TacM (N = 167)	P value
**Male, n (%)**	105 (55.0)	91 (54.5)	0.93
**Age, years ± SD**	50.37 ± 13.7	50.5 ± 13.5	0.70
**Caucasian n (%)**	174 (94.1)	149 (90.9)	0.26
**HLA mismatch AB, mean ± SD**	1.69 ± 1.07	1.93 ± 1.23	0.29
**HLA mismatch DR, mean ± SD**	0.74 ± 0.64	0.90 ± 0.66	0.17
**Induction, n (%)**			0.87
ATG	2 (1.1)	1 (0.6)	
Basiliximab	185 (97.4)	161 (98.2)	
Other	3 (1.6)	2 (1.2)	
**Primary disease, n (%)**			0.005
Primary glomerular disease/glomerulonephritis	57 (29.8)	43 (25.7)	
Cystic kidney disease	35 (18.3)	24 (14.4)	
Renovascular disease/ hypertension	20 (10.5)	20 (12)	
Diabetic nephropathy	19 (9.9)	11 (6.6)	
Tubulo-interstitial nephritis	5 (2.6)	0 (0)	
Urological complications	11 (5.8)	3 (1.8)	
Other	44 (23)	66 (39.5)	
**First transplantation, n (%)**	160 (83.8)	141 (84.4)	0.86
**Donor characteristics**			
**Male, n (%)**	90 (47.1)	87 (52.1)	0.35
**Age, years ± SD**	51.3 ± 12.0	49.8 ± 13.5	0.06
**Type of transplantation, n (%)**			0.07
Living	85 (44.5)	87 (52.1)	
Donation after brain death	71 (37.2)	43 (25.7)	
Donation after cardiac death	35 (18.3)	37 (22.2)	
**Cold ischemia time, deceased donors only, min ± S.D.**	478.7 ± 502.5	409.1 ± 457.5	0.04
**Delayed Graft function,n (%)**	44 (23.0)	41 (24.6)	0.74

Chi-squared test was used for the donor and recipient baseline characteristics. CsA: cyclosporine A with or without prednisone and MPS or MMF, Tac: tacrolimus with or without prednisone and MPS or MMF.

### BKPyV infection and BKPyVAN

A total of 358 patients were included in the analysis with 78 tested positive for BKPyV-DNA in plasma in a range from 2 log10 to 7.17 log10cp/ml (Table 2). In one patient no samples were available at the defined time points. From the other 77 patients at least one plasma sample was available at one defined time point. The BKPyV-load could be measured in 655 plasma samples (68.6%) from the CsAM group and 479 plasma samples (57.4%) from the TacM group, at any defined time point. In total 656 plasma samples were missing for measuring the BKPyV-load at the different time points (Fig 1).

**Table 2:** Proportion BKPyV viremia.

	6 weeks (p = 0.45)		3 month (p = 0.68)		6 month (p = 0.02)		12 month (p = 0.015)		24 month (p = 0.08)	
	CsAM n = 41	TacM n = 30	CsAM n = 41	TacM n = 31	CsAM n = 37	TacM n = 29	CsAM n = 34	TacM n = 32	CsAM n = 23	TacM n = 24
0–2 log10 cp/ml, n (%)	33 (80.5)	27 (90.0)	18 (43.9)	17 (54.8)	19 (51.4)	11 (37.9)	25 (73.5)	17 (53.1)	19 (82.6)	14 (58.3)
2–3 log10 cp/ml, n (%)	5 (12.2)	1 (3.3)	5 (12.2)	3 (9.7)	10 (27.0)	3 (10.3)	7 (20.6)	3 (9.4)	4 (17.4)	4 (16.7)
3–4 log10 cp/ml, n (%)	2 (4.9)	2 (6.7)	11 (26.8)	5 (16.1)	5 (13.5)	4 (13.8)	1 (2.9)	3 (9.4)	0 (0.0)	4 (16.7)
>4 log10 cp/ml, n (%)	1 (2.4)	0 (0.0)	7 (17.1)	6 (19.4)	3 (8.1)	11 (37.9)	1 (2.9)	9 (28.1)	0 (0.0)	2 (8.3)

Chi squared test was used for the proportion of BKPyV viremia at the different time points between the CsA and Tac treatment group.

The occurrence of BKPyV-viremia between the CsAM (n = 42, 22.0%) and the TacM group (n = 36, 21.6%) was comparable (p = 0.9). Yet, BKPyV-loads were significantly higher in the Tac group at time point 6 months and 12 months (Table 2). At time point 3 and 12 months no significant differences were found within the Tac or CsA group in occurrences of BKPyV viremia/ BKVPyVAN in patients with higher trough levels compared with patients with trough levels lower than median (Table 3).

**Table 3:** Proportion BKPyV viremia and BKPyVAN based on median trough levels.

**3 months**	**Tac median >9.5ug/l**	**Tac median < 9.5ug/l**	**P value**	**CsA median >183.5ug/l**	**CsA median <183.5ug/l**	**P value**
**BKPyV viremia, n (%)**	20 (50)	20 (50)	1	21 (56.8)	16 (43.2)	0.35
**BKPyVAN, n (%)**	6 (54,5)	5 (45,5)	0.76	3 (75)	1 (25)	0.31
**12 months**	**Tac median >8.4ug/l**	**Tac median <8.4ug/l**	**P value**	**CsA median >163.5ug/l**	**CsA median <163.5ug/l**	**P value**
**BKPyV viremia, n (%)**	14 (40)	21 (60)	0.16	20 (60.6)	13 (39.4)	0.17
**BKPyVAN, n (%)**	3 (50)	3 (50)	0.98	3 (60)	2 (40)	0.65

Chi squared tests were used to compare the proportion of BKPyV viremia and BKPyVAN in patients with trough levels higher and lower than median at 3 months and 12 months after renal transplantation.

**Fig 4:**
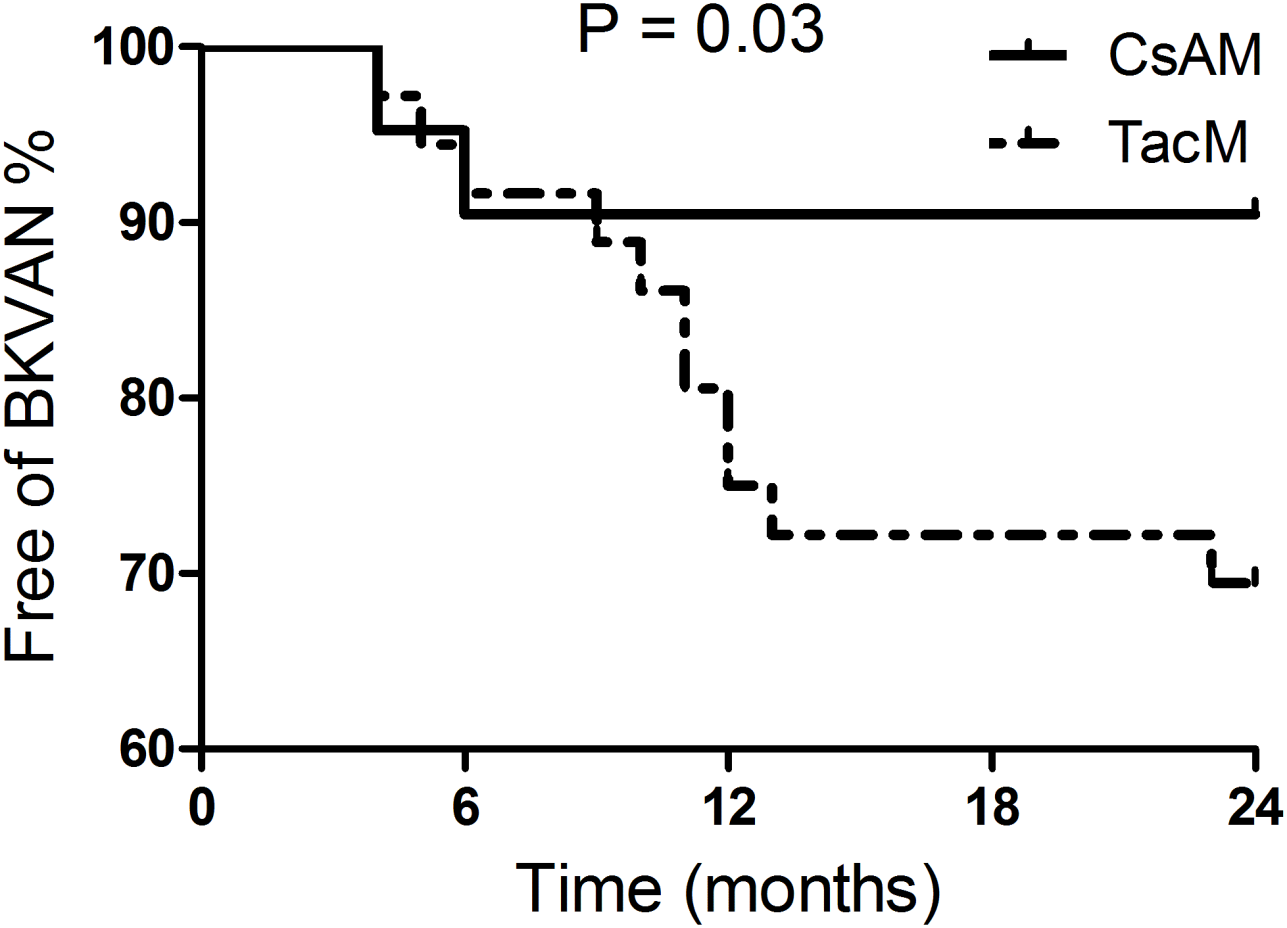
Kaplan-Meier BKPyV nephropathy in time. There was a significant difference in the incidence of BKPyVAN over 24 months between the two treatment groups (p = 0.03). CsAM: cyclosporine A with MPS or MMF, TacM: tacrolimus with MPS or MMF. CsAM: cyclosporine A with or without prednisone and MPS or MMF, TacM: tacrolimus with or without prednisone and MPS or MMF, BKPyVAN: BK polyomavirus nephropathy, BPAR: Biopsy proven acute rejection.

In total 15 patients developed BKPyVAN (4.2% of the total cohort). Of these patients, four (2.1%) were treated with CsAM and eleven (6.6%) with TacM (p = 0.03). In three of the four patients treated with CsAM, BKPyVAN occurred after treatment with anti- rejection therapy (methylprednisolone and ATG). In patients treated with TacM, two of the eleven were treated with anti-rejection therapy (ATG), before BKPyVAN was diagnosed. Fig 4 shows the occurrence of biopsy proven BKPyVAN over 24 months.

In 5 recipients with BKPyVAN, treatment was adjusted by either dose reduction of MPA, or switching from Tac to another immunosuppressive. In 3 of these 5 recipients, BKPyVAN was diagnosed before any adjustment in immunosuppression was done (S1 Fig).

The BKPyV seroprevalence of the total population was 97.5%. In the RTR, who developed BKPyV-viremia, 76 were BKPyV-IgG seropositive before transplantation and two BKPyV-IgG seronegative. One of whom developed BKPyVAN. The IgG-titer, of the recipients, before transplantation was not significantly different between recipients with BKPyV-viremia (12128±8456) vs. no BKPyV-viremia (13935±8301) (p = 0.9).

Furthermore, in both treatment groups (CsAM and TacM) BKPyV genotype I was the most prevalent followed by genotype IV (p = 0.48). In recipients with BKPyVAN, genotype II had a higher prevalence than those with only BKPyV-viremia (p = 0.03).

### Course of BKPyV replication

**Fig 5:**
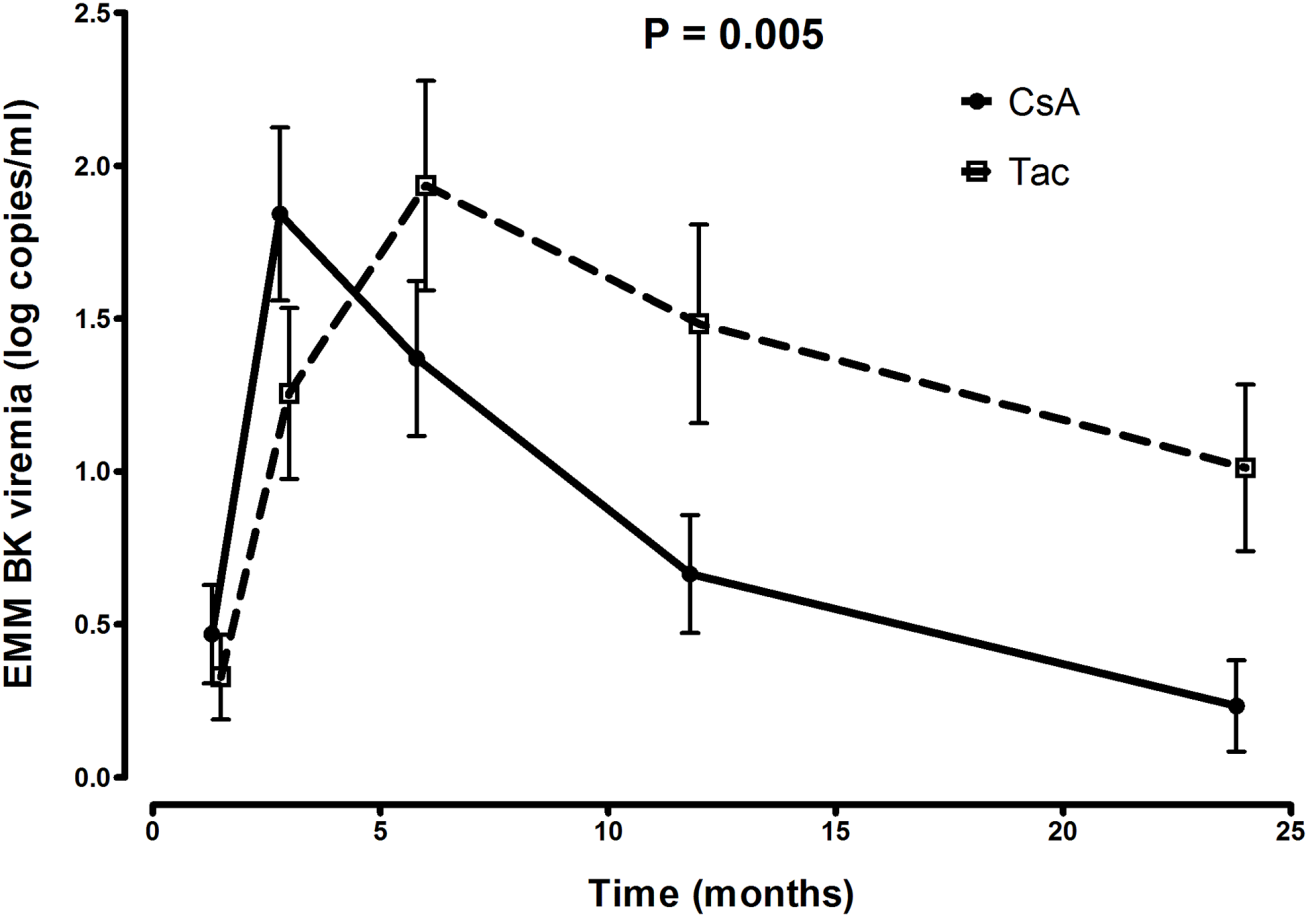
Longitudinal course of BKPyV viremia over 24 months. CsA: cyclosporine A with or without prednisone and MPA, Tac: tacrolimus with or without prednisone and MPS or MMF. Recipients in the CsA group had an earlier decline in BKPyV viral load (copies/ml) over the period t = 6 to t = 24 months (p = 0.005). GEE with an exchangeable correlation structure were used for calculating the P values.

Longitudinal data analysis showed a significant earlier decline of BKPyV-load, after tapering of immunosuppression, in plasma in the CsAM group (t = 3 months) compared to the TacM group (t = 6 months) (Fig 5). After adjustment for age, sex, donor age, donor sex, primary disease, type of donor (living,deceased), cold ischemia time, HLA mismatches (AB, DR) and BPAR it remained significant.

The AUC for BKPyV-load in RTR treated with CsAM was significantly lower compared to the TacM group (10.8±15.3 CsAM, 28±29.8 TacM) (p = <0.001).

### Associations with BKPyVAN

Due to the large number of factors relative to the sample size of recipients with BKPyVAN, several models were constructed with different donor and recipient transplant characteristics (recipient sex and age, donor sex and age, BPAR, cold ischemia time, HLA mismatch (AB, DR), primary disease, type of donor (living,deceased).

The Cox regression model showed a strong association of BKPyVAN for treatment with TacM (hazard ratio = 3.38, 95%CI = 1.075–10.61) which became even stronger after adjustment for donor type (living vs. deceased), cold ischemia time and BPAR (hazard ratio = 4.29, 95%CI = 1.307–14.05) (Table 4).

**Table 4:** Association between immunosuppression and BKPyVAN.

	Hazard ratio (Exp B)	95% CI		P value
Crude model	3.378	1.075	10.61	0.037
Model 1	4.286	1.307	14.05	0.016
Model 2	3.636	1.125	11.750	0.031
Model 3	3.365	1.067	10.609	0.038
Model 4	3.428	1.083	10.849	0.036
* Crude model	3.741	1.190	11.762	0.024
Model 5	3.897	1.223	12.413	0.021

Cox regression was used for multivariate analysis of proven BKPyVAN during 24 months, between the treatment arm TacM vs. CsAM (reference). *Model 1*: adjusted for type of donor (Living vs. deceased), cold ischemia time and BPAR. *Model 2*: adjusted for HLA mismatch (AB, DR). *Model 3*: adjusted for primary disease. *Model 4*: adjusted for recipient sex and age. *Model 5*: adjusted for donor sex and age.

* The crude model of model 5 differs from the other models in the number of missing cases.

### Effect of BKPyV infection on renal function

**Fig 6:**
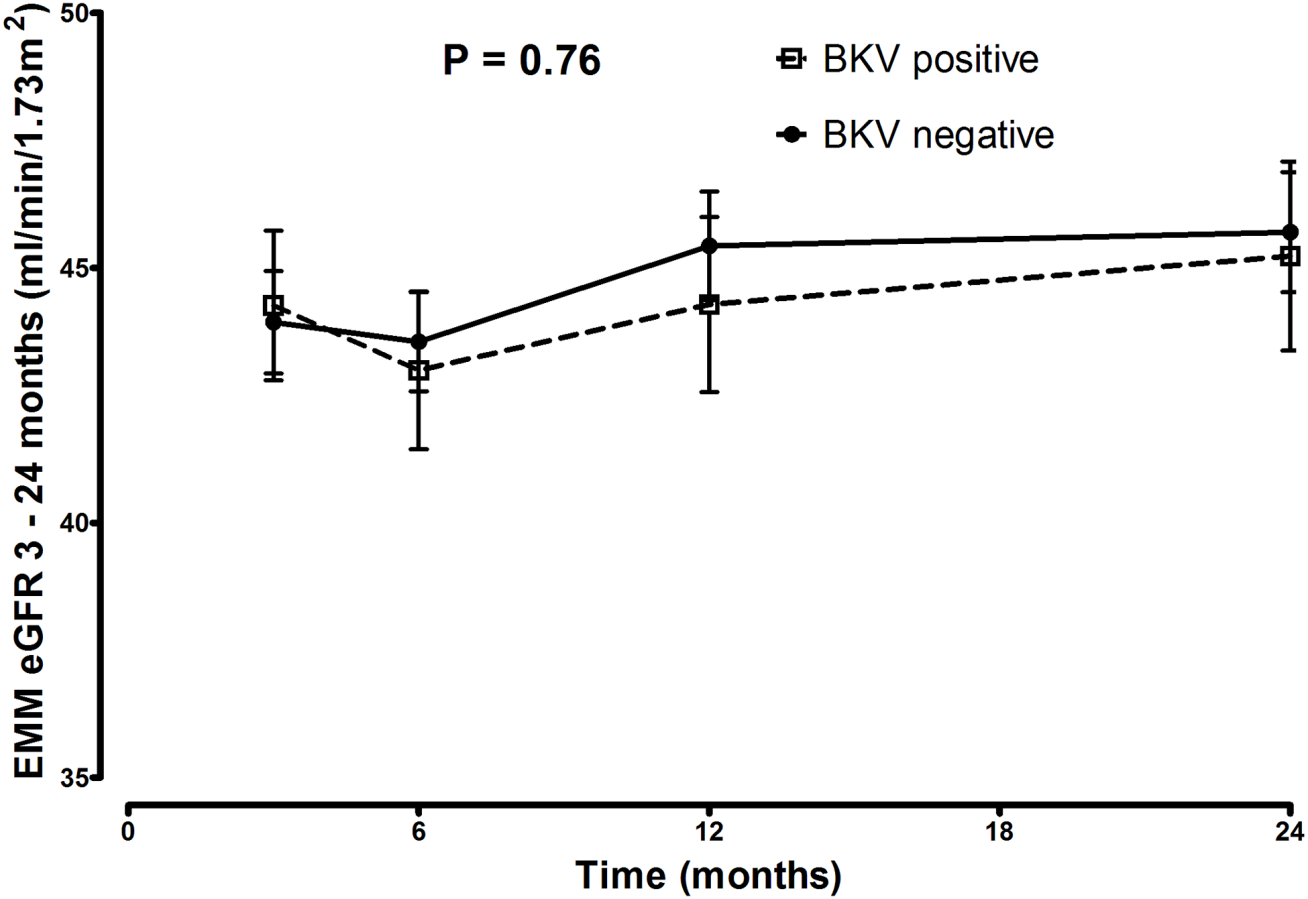
eGFR between 3 and 24 months in BKPyV group. The eGFR is not significantly different over 24 months between recipients in the BKPyV positive group (recipients with viremia) vs. BKPyV negative group (recipients without viremia) (p = 0.76).

In order to determine the effect of BKPyV replication on the renal function, eGFR rates and the longitudinal course of eGFR between the BKPyV negative and plasma BKPyV positive group were compared, over 24 months. No significant difference was found between the groups in mean eGFR at t = 24 months (46.8mL/min/1.73m^2^ and 47.8mL/min/1.73m^2^ respectively) (p = 0.88). Also the longitudinal course of eGFR did not differ in both groups, as depicted in Fig 6.

**Fig 7:**
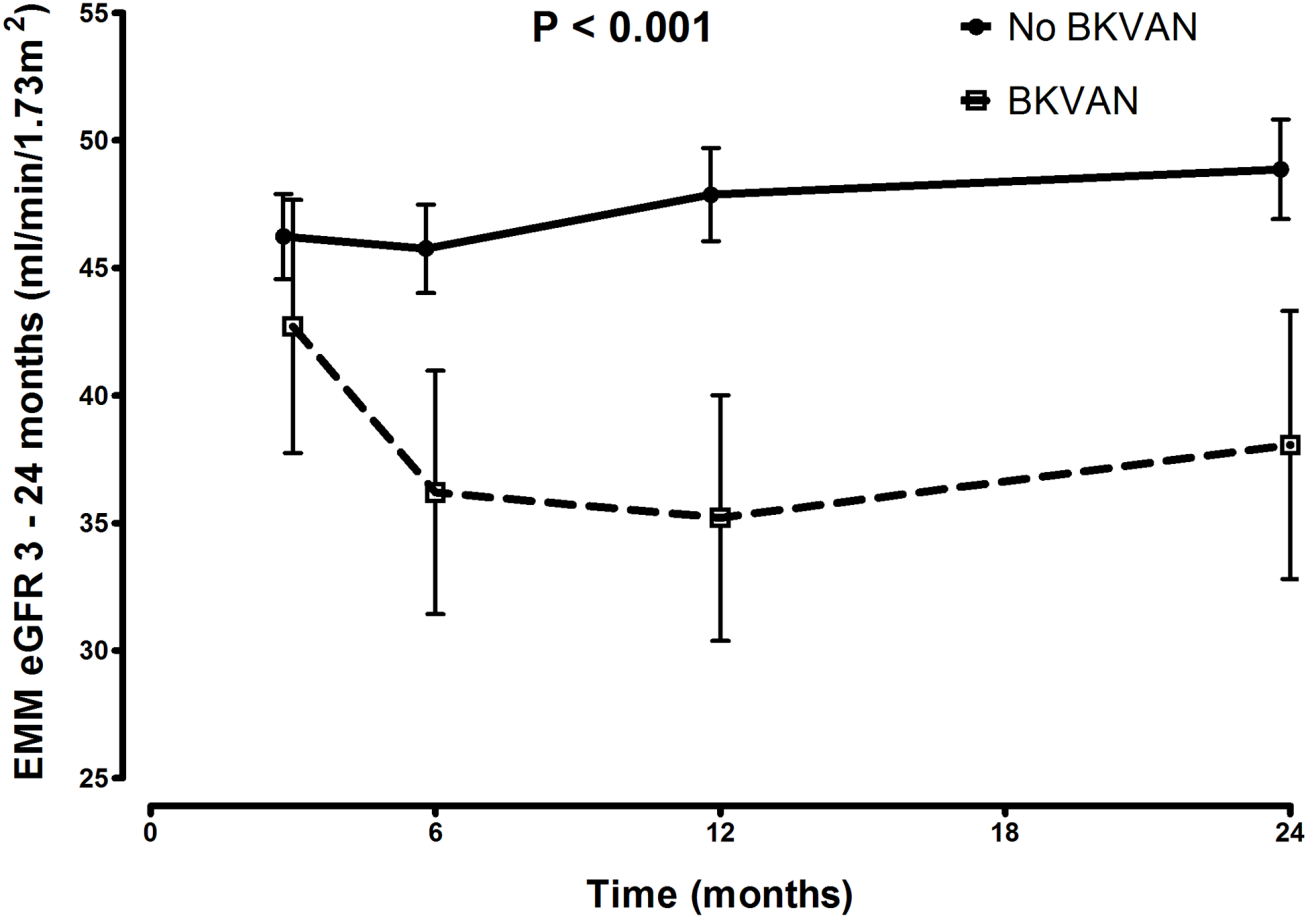
eGFR between 3 and 24 months in BKPyVAN group. eGFR over 24 months is significantly lower in recipients with BKPyVAN (p = <0.001).

EMM of eGFR rates was significantly lower in the group with BKPyVAN (Fig 7). It remained significant after adjustment for age, sex, donor age, donor sex, donor type (living,deceased), primary disease, HLA mismatches (AB and DR), cold ischemia time and BPAR.

### Secondary outcomes: Death, graft loss and BPAR

Graft loss and mortality rate at t = 24 month were comparable in both treatment groups (CsAM vs. TacM) (Table 5) and in BKPyV positive vs. BKPyV negative group (data not shown). BPAR occurred more frequently in the CsAM (19.5%) group compared to the TacM (10.8%) (p = 0.02). BPAR occurred mostly before adjustment of treatment and in seven patients (50%) before BKPyV viremia was diagnosed (S1 Fig).

**Table 5:** Secondary outcome over 24 month period.

	CsAM (N = 191)	TacM (N = 167)	P value
**Death, n (%)**	15 (7.9)	9 (5.4)	0.35
**Graft loss, n (%)**	14 (7.3)	12 (7.2)	0.96
**BPAR, n (%)**	38 (19.9)	18 (10.8)	0.02

Chi-square test was used for the efficacy related results over 24 months between the treatment groups. CsA: cyclosporine A with or without prednisone and MPS or MMF, Tac: tacrolimus with or without prednisone and MPS or MMF.

The renal function of RTR with BPAR vs. no BPAR showed a tendency to difference (p = 0.067) (S2 Fig).

## Discussion

Several risk factors have been described for developing BKPyV replication and BKPyVAN in RTR, and it is well known that immunosuppression plays an important role [18–20]. It is unclear whether the overall intensity or the combination of specific immunosuppression has an effect on the occurrence of BKPyV complications.

In this retrospective cohort study, we investigated the effect of immunosuppressive treatment with MPA and two different calcineurin inhibitors, CsA and Tac, on the frequency of BKPyV viremia and BKPyVAN in kidney transplant recipients.

An interesting finding was the incidence of BKPyV-viremia. Although this was comparable in both treatment arms, CsAM therapy was associated with an earlier decline and lower total BKPyV-load than TacM therapy. Also, the occurrence of BKPyVAN was lower in the CsAM group, at the cost of increased BPAR. These findings could possibly be ascribed to the relative high drug levels of Tac at month 3 to 12, as it is known that treatment with Tac is a risk factor for BKPyVAN. For this, patients were stratified according to the median measured trough levels at 3 and 12 months. In this study, however, no difference could be observed in incidence of BKPyV-viremia or nephropathy between the groups with low and high trough levels for Tac. In addition, CsA treatment is related with higher rates of BPAR and the role of BPAR, as a risk factor for BKPyV, is stressed, especially in the CsA-treated patients. Furthermore, the combination of Tac with MPA results in more intense immunosuppression than CsA with the same dose of MPA, because the system exposure to MPA is up to two fold higher in patients receiving Tac versus CsA. This is due to CsA inhibition of the enterohepatic recirculation of the major metabolite MPA glucuronide [21,22].

Our finding of the earlier clearance of BKPyV by treatment with CsAM is in line with results of Hirsch and colleagues [1]. In their study, including 682 RTR, lower rates of viremia at 6 and 12 months were observed in the CsA group compared to the Tac group. The study of Mengel et al also showed higher rates of BKPyVAN in patients treated with Tac and MMF [11] compared to other clinical studies that showed increased risk of developing viremia, but no significant differences in the incidence of BKPyVAN between treatment groups with CsA and Tac [23,24] Our findings further strengthen the notion that the combination of MPA with CsA versus Tac results in differences in the course of BKPyV replication after kidney transplantation.

Previously, the landmark ELITE-symphony study, including an immunological low-risk population, showed that treatment containing Tac resolved in less BPAR after 12 months, but occurrence of BKPyV-viremia and nephropathy were not reported [25]. Since the outcome in the first two years’ post-transplant overall survival and renal function are similar in treatment with either CsAM or TacM, the choice of immunosuppression pre-and post-transplantation should be made while considering the recipients risk profile, for allograft rejection or BKPyVAN. Calculation of the Tac metabolism rate as reported recently, might help evaluating the risk for BKPyV infection after renal transplantation [26]. In addition, in RTR with BKPyVAN switching from Tac to CsA could be considered, as our data suggest a more rapid clearance of BKPyV. The antiviral properties of mTOR inhibitors should be considered as an alternative in case of low immunological risk [27,28]. Additionally, in this study no predictive value of BKPyV serology of the recipient’s pre-transplant was found. Yet others have shown that high BKPyV-IgG titers in the donor and low BKPyV-IgG titers in the recipient, have a negative effect on the BKPyV infection post transplantation [29–31].

In this study we found genotype I to be the most prevalent. An interesting finding was that BKPyV genotype II had a higher prevalence in recipients developing BKPyVAN compared to recipients with only viremia. In the viremia group genotype IV was more prevalent after genotype I. This outcome differs from the one recently published by Schwarz et al. where genotype IV was more prevalent in the BKPyVAN group [32]. This discrepancy could possibly be explained to the different distribution of BKPyV genotypes in the population throughout the world, as described by others [33–35].

The present study has several limitations. As we did not perform AUCs of MPA we cannot exclude this effect, possibly contributing to the observed differences in both BKPyV associated complications and the occurrence of BPAR. This means we cannot conclude whether the observed differences result from the isolated immunosuppressive effects of Tac or the combined effect of Tac and MPA.

Secondly, patients were grouped according to the immunosuppressive treatment initiated directly after transplantation. Subsequent switch of immunosuppressive treatment was not included in the analysis. However, our data show that in the CsAM group most patients switched or stopped with CsA after rejection. In the TacM group the same was observed for BKPyVAN. This strengthens our findings that immunosuppressive regimens consisting of Tac is associated with a higher incidence of BKPyVAN and regimens with CsA more episodes of BPAR. Nevertheless, our results are in line with the literature and are derived from real time clinical practice, making them relevant for transplant care.

The effect of these two calcineurin inhibitors could be assigned to the different influence on the cell mechanism, which could affect the progress of BKPyV replication. Egli and colleagues showed that CsA and also Tac can cause a dose dependent inhibition of BKPyV specific IFN-gamma response of T-cells, whereas MPA, leflunomide and sirolomus did not [36]. In contrast, a few *in-vitro* studies have shown that CsA suppresses viral replication of different viral infections, including BKPyV [9,37–40]. It is not known whether these effects are the same *in-vivo* or if other interactions influences the drug mechanisms.

The current treatment for BKPyVAN is reduction of immunosuppressive agents, using BKPyV plasma loads for follow up. Several studies, have shown that switching to a mammalian target of rapamycin (mTOR) inhibitor is effective through the antiviral capacity of these inhibitors [41–44]. Recently, Hirsch demonstrated that BKPyV replication is activated by Tac and inhibited by the mTOR inhibitor sirolimus using a pathway involving FKBP-12 [28]. To gain more insight in the management and usage of immunosuppression, future studies should focus on the biological mechanisms and interactions of the allograft and viruses with the immune system.

In conclusion, this study demonstrated that immunosuppressive treatment with Tac and MPA is associated with a lower incidence of BPAR, but at the cost of an increased risk of developing BKPyVAN in the first two years’ post-transplant. Overall transplant survival was similar, which makes the choice of immunosuppression a delicate balance between BKPyV infection and BPAR.

## Supporting information

S1 Fig Overview switch immunosuppressive. Overview of recipients that switched from the treatment group directly initiated after transplantation. The figure shows how many recipients had a diagnosis BKVAN or BPAR and if this occurred before or after switch in treatment. CsAM: cyclosporine A with or without prednisone and MPS or MMF, TacM: tacrolimus with or without prednisone and MPS or MMF, BKVAN: BKV nephropathy, BPAR: Biopsy proven acute rejection.
(TIF)

S2 Fig eGFR between 3 and 24 months. Over the time period t = 3 to 24 months eGFR differs not significantly between recipients with BPAR vs. recipients with no BPAR (p = 0.067).
(TIF)
