# Supplementary figures and images for: A delicate balance between rejection and BK polyomavirus associated nephropathy; A retrospective cohort study in renal transplant recipients
Source: PLoS One. 2017 Jun 13;12(6):e0178801. doi: 10.1371/journal.pone.0178801 (PMC5469458; doi:10.1371/journal.pone.0178801)

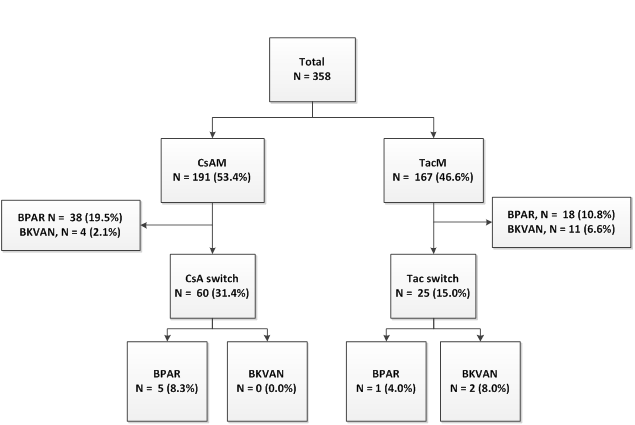

Supplement: S1 Fig — Overview of recipients that switched from the treatment group directly initiated after transplantation. The figure shows how many recipients had a diagnosis BKVAN or BPAR and if this occurred before or after switch in treatment. CsAM: cyclosporine A with or without prednisone and MPS or MMF, TacM: tacrolimus with or without prednisone and MPS or MMF, BKVAN: BKV nephropathy, BPAR: Biopsy proven acute rejection. (TIF) [file pone.0178801.s001.tif]

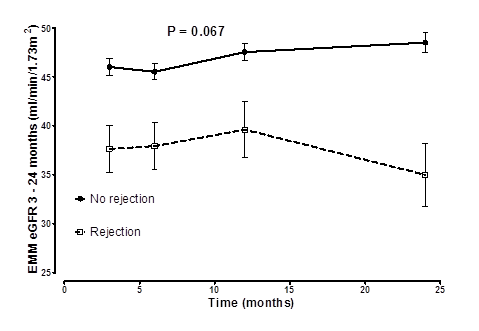

Supplement: S2 Fig — Over the time period t = 3 to 24 months eGFR differs not significantly between recipients with BPAR vs. recipients with no BPAR (p = 0.067). (TIF) [file pone.0178801.s002.tif]
